# Channels with Helical Modulation Display Stereospecific Sensitivity for Chiral Superstructures

**DOI:** 10.3390/polym13213726

**Published:** 2021-10-28

**Authors:** Renáta Rusková, Dušan Račko

**Affiliations:** 1 Polymer Institute, Slovak Academy of Sciences, Dúbravská Cesta 3, 84541 Bratislava, Slovakia; renata.ruskova@savba.sk; 2 Department of Plastics, Rubber and Fibres (IPM FCFT), Faculty of Chemical and Food Technology, Slovak University of Technology, Radlinského 9, 81237 Bratislava, Slovakia

**Keywords:** polymer, knot, topology, chirality, DNA, molecular dynamics, coarse-grained simulations, confinement, nanochannel, nanotechnology

## Abstract

By means of coarse-grained molecular dynamics simulations, we explore chiral sensitivity of confining spaces modelled as helical channels to chiral superstructures represented by polymer knots. The simulations show that helical channels exhibit stereosensitivity to chiral knots localized on linear chains by effect of external pulling force and also to knots embedded on circular chains. The magnitude of the stereoselective effect is stronger for torus knots, the effect is weaker in the case of twist knots, and amphichiral knots do exhibit no chiral effects. The magnitude of the effect can be tuned by the so-far investigated radius of the helix, the pitch of the helix and the strength of the pulling force. The model is aimed to simulate and address a range of practical situations that may occur in experimental settings such as designing of nanotechnological devices for the detection of topological state of molecules, preparation of new gels with tailor made stereoselective properties, or diffusion of knotted DNA in biological conditions.

## 1. Introduction

Polymers are long molecules consisting of many repeating units [1]. Different polymers are defined by the chemical composition of their building units, but polymers can also be defined by their architecture, i.e., how the building units are connected. The polymer architecture defines molecular landscape or topology [2]. While different architectures such as branched polymers [3,4], polymer stars [5], comb polymers [6], cyclic polymers [7], etc., have been relatively well explored, a new group of topological polymers is represented by polymer knots [8]. Control of molecular topology poses additional challenges to synthesis, separation and analytical methods for the detecting and separation of molecules with a defined topology. Molecular knots with well-defined properties can be prepared in limited amounts thanks to progress in synthetic methods [8,9]. A relatively straightforward approach to preparing polymer knots is linking the polymer ends in a melt of linear chains by cyclization relying on randomness of polymer coiling producing knotted chains probabilistically [10]. However, the separation of knots with desired topology from such melt would be virtually impossible with the current state of the art in analytical methods. Besides artificial polymers, natural sources of knotted polymers are DNA and proteins, where by far the most prevalent knot type is the simplest of knots—a trefoil, as well as knots with more complex topology were discovered [11,12,13]. The DNA knots were first discovered in 1976 [14]. These knots form naturally as result of action of molecular machinery, such as topoisomerases and recombinases [14,15,16]. Here, the most common bulk technique for identifying the DNA knot complexity and molecular knots’ topology is the gel electrophoresis [17]. In addition to the electrophoresis, DNA is often a subject of single-molecular experiments [18,19]. Here, a several techniques are developed to induce knots to the DNA chain by a number of approaches such as electric fields [20], mechanical tweezers [21,22], enzymes [15,16], cyclization [11], etc. In single-molecular experiments, the topological state of molecules is detected by nanotechnological devices where the knotted portion is driven through narrow channels or pores [23].

Another topological property of molecules that has posed long-standing challenges to its control is molecular chirality. Even after more than 150 years after the discovery of biomolecular chirality [24], the common methods for obtaining well-defined representatives of chiral pairs—enantiomers—are by leading synthesis in a certain way, what happens well in vivo; however, it brings a price of increased costs of synthesis in organic chemistry or by recrystallization which is not always feasible if the enantiomers do not form crystal units. The challenges of controlling the chirality are virtually identical with those connected to obtaining well-defined molecular knots [25,26]. There are more common features relating knottedness [27] (sometimes also called knottiness [28])—the state of being knotted—and chirality. First of all, knottedness and chirality are intrinsically bound [29]. The majority of knots are chiral [30]. However, not all knotted molecules are chiral, and not all chiral molecules are knotted. For a group of knots with increasing complexity characterized by a crossing number up to 11, there exists in total 801 prime knots [31], out of which 20 are achiral or amphichiral knots [32]. Polymers can consist of building units that are chiral, and in the case of DNA, the DNA is intrinsically chiral, forming a right-handed double helix from two DNA strands wound “negatively” around each other in anticlockwise direction [33]. Knottedness and chirality are not confined in a particular length scale, and they freely spread through smallest of existing scales over macroworld [34].

It is in our current knowledge that the topological state of molecules has important consequences on the physical and biophysical manifestations of properties of molecular systems. The behavior of circular molecules, which are topologically a ground-state zero of knottedness—known as unknots—shows distinct behavior from the linear chains such as slowing down of dynamics [35,36], where this effect increases with the complexity of polymer knots [37]. In biological systems, undesired knottedness and wrong chirality can be detrimental. While hundreds of proteins have their polypeptide chains knotted in their native state [38,39], the knots on DNA are undesirable and have to be efficiently removed. Why some proteins are knotted is not yet well understood; however, it is known that knots on DNA can lead to a mechanical breakage of DNA and the obstruction of molecular machinery, such as removal of transcribed RNA [16,40]. The importance of controlling chirality for pharmacophores became notoriously known [41]. In the case of DNA that is by itself chiral [33], the twisting of DNA in the opposite direction of the DNA’s handedness can induce underwinding and melting of DNA resulting in R-loops and genetic malfunctioning [42]. Existing experiments revealed existing abundance of knots with certain chirality [43]. As DNA exists essentially in an unknotted state [44,45], the topological investigations of DNA knottedness are commonly conducted in a reverse way, i.e., how the DNA becomes unknotted. From this aspect, the abundance of a certain chiral form may mean the mechanisms for DNA unknotting are not chirally equal, possibly making one chiral form more problematic for the cell.

It is important to note that knots are mathematically defined as embeddings on closed spaces such as 1D curves [27]. On open curves, such as linear polymers the knot can be probabilistic, obtained by performing a direct closure that defines the knot [39]. In some cases, such as a knot on a stretched linear chain, the direct closure running between the stretched ends would lead to the cancellation of the knot that has driven efforts for development of more sophisticated approaches for determination of the topology. In a more robust approximation, the knots on open curves are treated as knotoids, where the occurrence of a knot with given complexity is obtained by projections of the closure into the surrounding space [46]. Then, the occurrence of the knot with a given complexity is given as a probability from all evaluated projections [47,48]. This picture is very prominent for open, i.e., more loose knots. The more precise methods for the evaluation of knots on open curves led to realization that the number of well-defined knotoids is larger than the number of knots on closed curves for a given number of crossings [46]. However, if the knots are localized and compacted, the closure and the given knot type prevalence is much more restrained and more straightforward for determination.

The localization of knots is a very important physical phenomenon related to polymer knots. The localization of knots on molecules is encountered in experiments where an external force is applied whether in a form of electrical field, the flow of surrounding media or mechanical pull applied on the ends of the polymer chain, in single-molecular experiments [21,49]. From early stages, knots start moving in a direction of the applied external force and eventually localize, while they continue drifting along the chain by a process called self-reptation, when the polymer chain involved in knotting slithers in a snake-like motion [21,49]. Later, the mechanism of self-reptation was revisited, and the additional explanation was proposed for knot movement by breathing of knot and exchanging portions of chain between knot and surrounding DNA [50]. The localization and drifting of knots have also very profound biological importance as they make a crucial part of our current understanding how the long DNA molecules get rid of knots and exist in unknotted state. Long molecules of polymers spontaneously create knots and probability of being knotted increases with the length of the polymer [10]. In addition, chromosomal DNA that is a very long polymer fiber formed from DNA and histones and should be essentially knotted due to the activity of topoisomerases that allow the passages of DNA strands through each other [51,52]. Several mechanisms proposed for how DNA gets rid of knots employing topoisomerases rely on the localization and drifting of knots whether by entropic forces, mechanical push by supercoiling or involvement of molecular machinery responsible for structure maintenance of chromosomes [53,54,55,56,57,58,59]. The size of localized knots, drift speeds and other properties of left-handed and right-handed knots should be perfectly mirrored for the both enantiomers and cannot be used to distinguish between the chiral forms [60]. On the other hand, biological experiments show the abundance of left-handed DNA knots over the right-handed ones as produced in vivo [43]. So far, the proposed mechanisms that result in the abundance of one enantiomer account for the involvement of writhing or stereospecific sensibility of topoisomerase IV to certain type of crossings but do not address the common mechanisms of DNA unknotting mentioned above and possible differences in mobility of enantiomers in chiral environment [61,62].

In addition to topological constraints, the physical properties of polymers are affected also by confinement [63,64]. The manifestations of topological state emphasize placing knotted polymer into confinement. A confined state may be encountered whether in experimental arrangements such as nanofluidic devices or emerge naturally, in the dense environment of living cells. In a simple case, a linear polymer possessing a knotted portion can be driven through a narrow opening in the process known as pore translocation in polymers [65]. This scenario is encountered in biological conditions when knotted proteins translocate through pores in the cell membrane [66] or if a knotted DNA exits viral capsids [67,68]. Pore translocation is also an important mechanism accounted for in future nanotechnological devices for DNA sequencing [65]. Currently, the experiments with artificial pores and nanosized slits and channels show to be successful for detecting topological state of molecules and knotted portions on DNA chains [23]. The pore translocation of a knotted polymer occurs in several stages, while it is observed by experiments and also by computer simulations that the knotted portion would at first tighten and localize at the opening of the pore, and then it enters and translocates through the confined space of the pore, while the translocation of the knotted portion would usually occur in the latter stages of the polymer translocation [23,69]. It is not clear whether the drifting of knots, while they are in confining spaces of the pore, plays a role or if the knotted portion translocates with polymer chain as one whole; however, other experiments using nanotechnological devices called knot factories were used to study the drifting of knots along DNA confined permanently in narrow nanochannels [70], and this process was explored also theoretically. In confinement, additionally to the localizing effect of the driving force pulling the knot through the channel, also confinement helps to stabilize the size of knotted portion of the polymer if the confinement is sufficiently small [71,72]. In the case of translocation of knots on circular closed polymer chains, the computational experiments showed translocation can occur in two modes, whether with a localized knot in the later stages of polymer translocation or the translocation of entanglements of larger knot [69]. Current experiments examined various groups of knots and showed the twist knots are more apt to act like stoppers that are more difficult to translocate [73]. However, the experiments did not focus chirality of knots and did not try to exploit the confinement by imposing stereospecificity of the channels of some kind.

Finally, gel electrophoresis is a common method for studying and separation of circular knotted chains, such as DNA knots [17,33,74]. In this case, the topological state of molecules is constrained, and the molecules are confined permanently in the channels of the gel. During agarose gel electrophoresis, the knotted DNA molecules are separated based on an empirically known difference of mobility of molecules arising from the different knotted state moving through the narrow channels of the gel or resin. Gel chromatography allows also for separating chiral enantiomers of knotted molecules, however for the cost of chemical and topological pretreatment of the molecules [43,75]. Here, the ends of a chain containing knotted portion are ligated, and a righthanded (negative) supercoiling is introduced by gyrases to the molecule. Then, the different enantiomers are separated based on their different mobility in the channels arising from different internal motional degrees of freedom, given by competition of the righthanded supercoiling with the given handedness of the knot [76].

In this paper, we investigate the behavior of knotted polymers in chiral confinement in terms of their mobility and geometrical measures. We employ coarse-grained molecular simulations, as these have proven to be useful in similar computational experiments for studying drifting of knots and translocation, polymer knotting in nanochannels, process of gel electrophoresis and investigating behavior of topological polymers [69,77,78,79,80]. We investigate both knotted linear polymers as well as knotted circular chains, together with such parameters as knot complexity, geometry of channels, size of knots, etc. The spread of the problems was enlarged intentionally to the point where we can show the problem of geometrical sorting of chiral molecules is nontrivial and more simulation work would be needed to fully understand the questions that emerged, while the primary goal of this paper, i.e., to demonstrate that the helical channels do exhibit selectivity on chiral superstructures such as DNA knots, is attained. In the first case, i.e., the knotted linear polymers, we induce the pulling force on knotted linear chains while keeping one end of the polymer chain tethered, while we investigate if the confinement is capable of inducing different drift speeds of the knots along the chain. This model setup addresses practical situations such as a knotted portion on linear chain translocating through a narrow channel in nanotechnological devices developed for detection of topological state of molecules. These devices currently cannot distinguish the chiral state of the knotted portions; however, as our computer simulations infer, channels upgraded with a helical modulation would allow one to make these devices stereospecifically sensitive. The situation of a knot drifting along the chain in a chiral environment could also address the biological mechanism of DNA unknotting, when the knotted portion is pushed in chiral environment formed by densely packed righthanded plectonemes as found in organization of bacterial chromosomes [81] or that one can imagine in simplicity as an array of plectonemic nanoposts [82]. Additionally, we investigated the case of circular knotted chains, and we show that the stereospecific effect induced by the helical modulation of channel would be sufficient for the sensing of knot chirality and separation of knot enantiomers in gel electrophoresis. The methods for preparation of tailor-made helical channels with finely tuned chiral properties have already been devised for stereospecific catalysis but also for the stereospecific separation of small molecules of pharmaceutical drugs [83]. These methods belong to the emerging field of chiral nanotechnology [84]. Moreover, the current methods for nanochannel fabrication allow for creating channels with any geometry one may think of or aiming there [85]. The idea that we also test is that in the case of chiral superstructures such as polymer knots, the helical channels do not have to be carved so perfectly because the chiral superstructures represented by polymer knots are soft and flexible; hence, they can adjust to dimensions of the channel.

## 2. Materials and Methods

### 2.1. Model of DNA Chain

We performed coarse-grained molecular simulations of polymer confined in narrow chiral channels. The simulations were performed using Extensible Simulation Package for Research on Soft Matter (Stuttgart, Germany) [86,87]. As a model system for simulations we chose DNA bio-polymer. For this purpose, we constructed a polymer chain, consisting of beads connected by bonded potentials and having the excluded volume interaction. The size of the beads in the simulation is 1 σ. In physical units, it corresponds to the size of a chunk of DNA consisting of 10 base-pairs. Each bead thus has a physical width of 3 nm [55,73]. The beads are connected with bonded potentials representing covalent bonds and angle bending imposing stiffness to the chain. The covalent bonds were modelled by the harmonic potential *U*_s_(*r*) = *k*_s_(*r* – *r*_0_)^2^, where *k*_s_ is the force penalty against bond stretching and *r*_0_ is the equilibrium bond length set to 1 σ. We kept the stretching of the bonds limited by imposing a high stretching constant *k*_s_ = 10 ε_0_, where ε_0_ = k_B_*T* in order to prevent artificial passages between parts of the DNA chain and undesired changes in topological state of the chain that is exposed to external forces by pulling and compression in confinement. The bending potential was modelled by harmonic interaction *U*_θ_ = ½*k*_θ_(*θ* – *θ*_0_)^2^, where the force constant *k* represents penalty against bending of the chain and equilibrium angle *θ*_0_ = π. The constant was set to *k*_θ_ = 15 σ/ε_0_ beads which corresponds to persistence length *P*_l_ = 45 nm. The persistence length is often set at 50 nm [88], but other values have also been reported ranging between 35 and 55 nm with an average value from different methods given as 45 nm [89]. In our work, we are not revisiting the persistence length of DNA; rather, we aim to keep the simulations minimalistic on computational time. The volume of the polymer chain was modelled by nonbonded excluded volume interaction represented by fully repulsive shifted and truncated Lennard-Jones potential *U*_ex_(*r*) = 4ε_0_[(σ/*r*)^12^ – (σ/*r*)^6^ + 0.25], if *r* < 2^1/6^σ and *U*_ex_(*r*) = 0 otherwise [90]. The DNA chain in majority of the simulations was considered to be nicked, i.e., not storing torsional stress; however, for purpose of the proof-of-principle, we performed also some simulations with non-nicked DNA (Figure S2). Here, we employed our earlier model that we used for simulations of supercoiled DNA with the description and parameters of the model provided elsewhere [55,91]. During the simulations, one end of the chain was tethered, and the external force responsible for pulling and drifting of knots was imposed directly to the beads with amplitude of *F*_ext_ = 0.05ε_0_/σ corresponding to 0.07 pN and direction away from the tethered end. When the knots reached one end of the chain, this end was tethered while the other one was released, and the direction of the pulling force was flipped. In this way, we exploited longer sampling of the knots’ geometrical measures and dynamical behavior in terms of drift speeds and diffusivities. We performed Langevin dynamics simulations with the equations of motions $m{\ddot{r}}_{i}=-\gamma m{\dot{r}}_{i}-\nabla U\left(r_{i}\right)+R\left(t\right){\left(2k_{B}Tm\gamma \right)}^{0.5}$, where *r_i_* denotes position vectors of *i*-th bead; ∇U is the force calculated from particle’s interactions; $\gamma m\dot{r}=\xi$*v_i_* expresses friction where γ = 2ξ/*m* = *τ*_LJ_^−1^ is damping constant in units of reciprocal time; and the last term represents the random kicking force of implicit media, where *R*(*t*) is a delta-correlated stationary Gaussian process. The equations of motion were integrated with a time step Δ*τ* = 0.01. The physical time unit after transformation into Stokes’ time corresponds to [*τ*] = 123 ns [73]. A single-molecular dynamics trajectory was obtained by performing 5 × 10^6^ iterations, corresponding to 6.15 ms, and we ran 5 repetitive runs saving configurations every 1000 iterations. In this way, we obtained 25,000 structures for analyses that provided stable statistical averages. 

### 2.2. Molecular Topology

The initial structures with a given topology of a knot were generated based on parametric equations and *p*, *q* parameters for torus knots [27]; in the case of twist knots, coordinates were downloaded from Knot Atlas website [92], and beads were added by interpolation by using our routine KnotFit developed earlier [91]. In the case of simulations of knotoids, or linear chains with a knotted portion, we arbitrarily interrupted one of the bonds as the topology of resulting knotoid should not be dependent on the site where a bond is cut. In order to pre-localize the knotted portion, we pre-stretched the chain by imposing a pulling force *F*_ext_ = 10 pN to the both ends. During all simulations, we continuously evaluated the topology of the knot by using KymoKnot [93] and Knoto-ID software [46]. Both computational tools are easy to implement into simulation scripts, while Knoto-ID provides more information on chirality of knots in terms of nomenclature adopted in the related work [46] and distinguishes larger numbers of knotoid types. However, as Knoto-ID is a more complex tool it performs more slowly; hence, we used KymoKnot primarily to check if the topology is maintained along the simulations evaluating the structure every 1 µs. For some practical quick-checks, we used implementation of Knoto-ID within KnotProt server [39]. 

It is important to note in context of nomenclature of chiral knots, when it comes to distinguishing of a knot from its chiral pair, the current tables are ambiguous and choice of a representative of the chiral pair is often inconsistent [94]. When identifying a knot, one usually compares it with the Rolfsen’s table [95], while the representative of the chiral pair in the table is often marked with a prefix “+” and the sign “−” is used for its mirror image. In this way, the first of the knots depicted in the Rolfsen table that happens to have negative crossing number is often found as +3_1_ knot, while the “+” sign has nothing to do with the handedness or writhe of a knot. In order to avoid confusion with the handedness, some authors decline from using (+) and (−) signs [46]. Instead, an indicator “m” standing for “mirror image” is used to distinguish chirality of a knot from that denoted in the table. For practical purposes, we distinguish between different knots based on handedness and writhe, indicated by <+> or <−> prefixes to the knot type. This nomenclature is consistent with another study which originally realized that the writhe is useful for distinguishing chirality of DNA knots [96], and in the new biologically meaningful table, the mean writhe should be used to identify knots’ chiral pairs [94]. In the case of torus knots, the handedness and writhe of the knots are defined by the handedness of winding of a chain around a surface of torus. In the case of twist knots, the situation is more complex; hence, we indicate prevalent handedness by the sign of computed mean writhe. In the case of achiral knots, the mirror image is the same knot; hence, we do not associate it with a prefix.

### 2.3. Channels

We modelled the confining channel explicitly using beads with the diameter of 1 σ. Channels with different helical geometries were examined within our simulations. The channels were modelled by stacking circles composed of beads on each other. In such an approach, the helicity can be controlled by a multiple of parameters such as pitch, roll, slide, tilt, shift, etc., which have also been used for modelling of DNA’s helicity [97]. In the simplest case, one may think of a twirl of circles displaced by a radial increment around a central axis of rotation, i.e., controlling pitch, slide and roll parameters. In such an approach, however, the cross section of the channel would change with the radius of a helix, *R*_H_. In order to maintain the cross-section independent on the radius of the helix, one has to stack the circles perpendicularly to the equation of helix, i.e., also controlling the tilt, tip and inclination of the stacked circles. When using perfect circles with radius *R*_ch_ that define the radius of the tube or the channel, the resulting object is a helical tube known as the Archimedean spiral. The equations and algorithm to generate the hollow helical tube are provided as a part of Supplementary Information, Figure S1. 

In order to incorporate the knotted chains into the helical tube, we partially implemented the protocol adopted from our previous work on segregation of DNA chains confined in channels [98]. At first, the chains were placed into a cylindrical channel with very large radius *R*_ch_ >> *R*_g_. We applied pulling force colinear with the main axis of the cylinder but with opposite directions at the ends of the chain. As we were pulling, we started also shrinking the confining cylinder and decreasing its radius. This approach would be sufficient for placing knotted chains into helical channels if the radius of the channel is larger or approximately of the size of the radius of helix, *R*_ch_ ≥ *R*_H_. For a more general protocol, when the radius of the helix can greatly exceed the radius of the channel, *R*_ch_ << *R*_H_, we adopted an additional step when the implicit cylindrical confinement was replaced by the explicitly modelled tube. Then, we started molding the helix by slowly changing the radius of the helix and shifting positions of the beads forming the confining channel (See Video S1). In this way, we were able to place the chains into any confining helix with any parameters *R*_ch_ and *R*_H_. During this process, one has to pay attention only to the distances between beads that should not exceed diameter of 1 σ. In our computer simulations, we investigated behavior of knots in helical channels in three scenarios: the handedness of the channel was the same as the handedness of knots (in terms of their mean writhe) that we named equichiral system; the handedness of knots and the channel is opposite; hence, we called it antichiral system. Finally, we also performed simulations of achiral knots in the helical channel. In order to investigate behavior of achiral knots in chiral channels, we performed simulations by confining 4_1_ and 6_3_ knots into lefthanded and righthanded helical channels. 

## 3. Results and Discussion

### 3.1. Preliminary Measures of Pulled Knots in Free Spaces Do Not Exhibit Chiral Differences

First of all, we started with simulations of knotted linear molecules without confinement, in a free space with implicit solvent present. In this step, we obtained a picture on the geometrical measures of the knots, since the geometry of channels should be of similar sizes as the average measures of the knot in order to induce the effect of confinement. As the crucial parameter, here we chose the size in terms of gyration radius, *R*_g_, of the simplest of knots—the trefoil—and its knotoid obtained after interrupting one of the bonds and stretching of the chain in order to localize the knot. This size determines the radius of the channel *R*_ch_, which was set to be smaller than the calculated *R*_g_ and was used as default value also in the simulations of more complex knots, which naturally show increased *R*_g_. As for the stretching of the chain, this was achieved by tethering one end of the chain and imposing a pulling force on all beads with a direction pointing away from the tethered point. The external pulling force employed in the simulations was *F*_ext_ = 0.05ε_0_/σ, which corresponds to 0.07 pN, and it is equivalent also to a magnitude of an electrostatic field *E* = 280 mV·cm^−1^ [73]. This force was chosen based on preliminary simulations and also because it is in the typical range of forces encountered in experiments moving in the pN’s range. Rather than tensioning both ends of the DNA chain by applying additional force of 10 pN, as in the experiments employing mechanical tweezers, we kept one end tethered while the chain became stretched in a direction colinear with the vector of applied force. At the same time, we observed that this setting was also sufficient to localize the knotted portion on the polymer. Under the influence of the external force, the knots also slithered, or drifted, away from the tethered bead. Once the knots reached the free end of the chain we tethered this end, released the other end and reversed the direction of the force. This was made for the purpose of saving computational time and to avoid using very long chains that would be bearable for simulating free chains; however, the very long chains would heavily increase the computational costs in latter simulations of the polymers in confinement. We adopted this approach also based on the previous computer experiments performed in alternating forcefields of AC currents, which showed that after switching direction of the force, the steady-state quickly re-establishes in short periods below 0.225 ms. In our simulations, the average time needed for a knot to reach the end of the chain consisting of 100 beads was 0.5 ms for a trefoil with 10 switches of direction of the pulling force over the total simulation time (6.15 ms). In the case of the 4_1_ achiral knot, the drift speed is slower, and hence, the period needed to switch the direction of the pulling force is longer, as the knot is more complex and it also belongs to a group of twist knots that are known to move slower than torus knots and are used as stoppers. We performed simulations *τ*_tot_ = 6.1 ms long, saving 5000 structures for analyses of structural properties of knotted parts in terms of gyration radius of knotted part *R*_g_ and length of the knotted portion *L*_k_, also evaluating the asphericity, *A*, and prolateness, *P* [99], which were summarized in Table 1 for several knots. 

Table 1 also contains topological parameters on the knots in terms of knot type with its name based on convention implemented by Knoto-ID software. There could be cases when knotoids share the same invariant, e.g., 4_1 and 11n_19 share the same Jones polynomial. This should not be surprising as there are no complete polynomial invariants. Knoto-ID reports correctly that it detects a nontrivial knot that could either be 4_1 or 11n_19 based on the polynomial that it evaluated. However, the user has to also check the number of crossings that structure has and determine which of the two is true. The additional check is performed by KymoKnot software. Nevertheless, we keep and report all knots detected by Knoto-ID as the software’s standard output, in Table 1. 

We include also writhe of the knot, Δ*Wr*, and the corresponding notation based on the proposed nomenclature based on the biological relevance, where the sign is obtained based on the writhe, which we find extremely useful in discussion of the knots in chiral confinements. The geometrical measures of the knotted cores were computed as averages over the frames and also over the trajectories, obtaining a set of 25,000 samples for statistically equilibrated averages. The data summarized in Table 1 show that the gyration radius *R*_g_ increases with the complexity of knot. At the same time, the length of the knot, *L*_k_, representing the contour length of the knotted core, also increases. The contour length of the trefoil knot obtained by our simulations was 80 nm. The contour length of the knotted regions is dependent mostly on the persistence length affecting the gyration radius of the polymer. The previous study indicates only a slight variation of the knot size on the external force. Hence, the difference from the value of the knotted portion observed in the previous should be attributed to the difference in persistence lengths applied. 

The calculated values of asphericity and prolateness show complex behavior consistently with what was reported in the previous study on knotted polygons [99]. As the previous study has shown, the shape of knotted polymers has a complex behavior that changes most dramatically in the case of short polymer knots. The parameter of asphericity indicates deviations from a perfectly spherical shape, while the smaller values of *A* indicate that approaching zero the knot is more spherical. In the case of prolateness, *P*, the negative values indicate oblateness or disk-like-shaped geometry and values approaching 1 correspond to cigar-like elongated objects. From the calculation of the geometrical parameters of selected knots summarized in Table 1, we see the behavior of *A* and *P* is complex; however, one may expect disk-like shapes for knotoids obtained from torus knots (3_1_, 5_1_, 7_1_ and 10_124_), and while more elongated cigar-like shapes are observed for twist knots (4_1_, 5_2_, and 6_1_). The obtained geometrical measures are mirrored, obtaining the same values within the range of standard error deviation for pairs of enantiomers.

**Figure 1:**
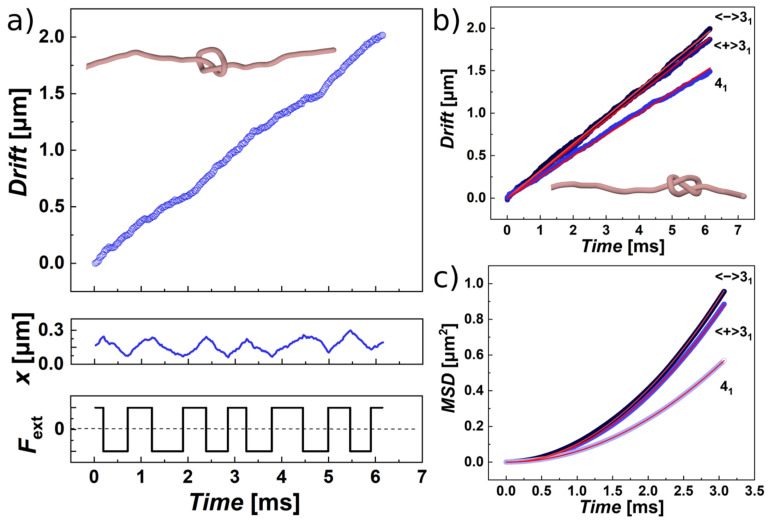
Drift analysis of knots along polymer chain from molecular trajectories. (**a**) The figure shows the distance Δ*x* travelled by the drifting knot as a function of time. A snapshot from the simulation shows the shape and localization of a trefoil knot, <+>3_1_. The graph in the middle shows the instantaneous position of the knot along the polymer, *x*. The sawtooth pattern emerges from the changes of the direction of external pulling force. The bottom graph shows the periods of pulling with positive or negative force *F*_ext_ = 0.05 ε_0_/σ = 0.07 pN oriented along the *x*-axis. (**b**) The graph shows drifted distance for selected knots (<+>3_1_, <−>3_1_ and 4_1_) with linear fits (red lines) with the slope corresponding to the drift speed. A snapshot from the simulation shows the shape and localization of the achiral 4_1_ twist knot. (**c**) The panel shows ballistic behavior of mean square displacement (*MSD*) typical for movement of particles in the presence of external force fitted by quadratic equation (0.05 × *Dt*/σ)^2^ + 2*Dt* (shown as red lines) in order to obtain diffusivities.

The drift speeds were computed from knot displacements Δ*x* along the polymer over the simulation time, averaged for five trajectories for each knot, by fitting linear lines with a slope corresponding to the drift speed *v*_drift_ and intercepting at zero. The displacement Δ*x* was obtained as the sum of instantaneous displacements computed from different frames in the trajectory multiplied by the direction of the force Δ*x* = sign (*F*_ext_) × (*x*_i + 1_ − *x*_i_), for *i* = 1,2 … *N*_frame_, where *N*_frame_ = 5000 is the total number of frames. Multiplication by sign (*F*_ext_) during summation was used for linearization of the drift, i.e., the total distance travelled by a drifting knot. The position of the knotted portion was obtained by analyzing the position of knotted core, giving also indexes of the starting and ending beads. The position of the knotted core was then determined in coordinates along the chain by calculating the central index of the knotted core, or it is also possible to calculate positions in terms of the center of mass of the knotted core. In computer simulations, both ways are equally feasible, and the results are highly correlated. In experimental settings, the coordinates of the confining channel were also used. The drift speeds with the standard error deviations were obtained by concatenated fits over the trajectories for a particular knot type. The drift of the sample knots 3_1_ and 4_1_ are shown in Figure 1 as a function of time along with actual positions of the knots. The instantaneous position of the knots under and alternating external force shows a sawtooth pattern as observed also in the earlier work [73]. The computed drift speeds from the simulations have a similar value for the knots from the same group, i.e., torus knots and twist knots show a difference in drift speeds from each other, while the computed drift speeds within the same group remain approximately the same. Comparison of the drift speeds in our computational setting shows the speeds higher than observed in the previous work. This difference most likely originates from the absence of the additional pulling force that would tighten the knots on the chain. The absence of the additional pulling force tightening the knots is measured also on diffusivities of the knots.

The previous experimental and computational works studied the diffusion of the knot along a DNA stretched by pulling applied at the both ends of the chain, and the diffusivity is computed from the mean square displacement of free Brownian motion of the knot along the chain [21,49,50,73,102]. In our experimental setting, the diffusivities have to be obtained in a different manner. We consider the computed mean square displacement (*MSD*) is a result of two coupled processes, self-diffusion and drifting induced by a pulling force. [103] The *MSD*’s curves under the influence of an external force exhibit a ballistic behavior with a shape of a quadratic function. The *MSD* can be described as *MSD* = (*v*_0_*t*)^2^ + 2*Dt* [103,104]. Here, the first term (*v*_0_*t*)^2^ describes movement by velocity *v*_0_ induced by external force, where *v*_0_ = *F*_ext_/γ_k_. The external force *F*_ext_ corresponds to the value 0.05ε_0_/σ = 0.07 pN, and γ_k_ is the friction related to the drifting of the knot along the polymer. The friction γ_k_ is related to self-diffusion of the knot along the DNA chain, *D* = ε_0_/σ_k_. Hence, the equation describing the mean square displacement can be rewritten in the form *MSD* = (0.05 × *Dt*/σ)^2^ + 2*Dt*. The second term in the equation 2*Dt* stands for the longitudinal diffusion colinear with the direction of the pulling force. The resulting equation is a quadratic equation that is fitted over the calculated *MSD*’s, which in the field of external force exhibit a ballistic dependence. The measured diffusivities are higher that previously determined, but the increase is not dramatic, and the values are consistent with the faster drift speeds for our particular model setting [21,49,50,73,102]. The measured diffusivities of knots along DNA chain have been shown to vary depending on experimental settings such as pulling force or chain sizes, while the values in the range between 1 and 12.5 mμ^2^·s^−1^ were reported for a trefoil [21,49,50,73,102]. In our simulations and the model setting, we observe the diffusivity of the right- and left-handed trefoils being 16 mμ^2^·s^−1^ (Table 1). The increased values of the measured diffusivities can be result of absent stretching force at the polymer ends, but they also may originate from the short length of polymers used, as pointed out previously that smaller diffusivities were observed on much longer DNA chains with length up to 100 microns. Additionally, we would like to note that the values of drift speeds and diffusivities in Table 1 were obtained for a single setting of the pulling force *F*_ext_ = 0.05ε_0_/σ = 0.07 pN. As the existing work suggests, the degree of localization of the knot with a given topology characterized in terms of frictional length does not affect the drift speeds [73]; thus, we suppose the external pulling force does not affect the diffusivities, and the measured higher values should be related mostly to the short length of polymer employed in our simulations. This assumption seems natural, as the polymer ends perform faster movements, and hence, in the case of short chains, the increasing proximity of the chain ends may increase the dynamics of the entire chain. The comparison of the dynamical parameters, *v*_drift_ and *D*, obtained from the simulations shows again that these parameters are mirrored and symmetric for the pair of enantiomers and cannot be used for distinguishing chiral forms.

### 3.2. Pulled Trefoil Knot in the Helical Confinement Shows Symmetry Breaking

In the next step, we placed the linear knotted polymer containing a knotted portion corresponding to a trefoil confirmed by Knoto-ID or a direct closure, into a helical channel. A helical channel can be characterized by its chirality and three additional parameters, diameter of the channel, *R*_ch_ = 3 σ = 9 nm, radius of the helix, *R*_H_, and the pitch, *k*; see Figure S1. The pitch is a parameter that characterizes elevation or distance of two loops given as 2π*k*. We chose the pitch such that the loops made from channels with radius *R*_ch_ = 3 σ sit on each other and do not extend into each other, i.e., *k* = 1.5 σ. The resulting period of the loops 2π*k* ≅ 28 nm is large enough to accommodate the trefoil knot with the *R*_g_ = 7.7 nm determined in the previous section and also all of the more complex knots investigated in Table 1. The value of the radius of the helix, *R*_H_ = 1.5 σ = 4.5 nm, was chosen arbitrarily from an intermediate range as we could not address a particular experimental setup. Since the radius of the helix is smaller than the radius of the channel, *R*_H_ < *R*_ch_, and the channel has a small curvature, κ. The curvature and the torsion of the helical tube are expressed by *κ* = *α*^2^/[*R*_H_(1 + *α*^2^)] and *τ* = α/[*R*_H_(1 + *α*^2^)], respectively [105]. The parameter α = ±*R*_H_/*k* is the subtended angle, and it defines the ratio between the curvature and the torsion. The chirality of the helix is given by the direction in which the circular cross-sections are rotated about the main axis of the symmetry. We use a negatively wound helix to address equichiral and antichiral scenarios, and we consider those being symmetric for a positively wound helix.

Naturally, in the first simulation setup, we modelled the simplest of knots—a trefoil. As mentioned above, we investigated two possible scenarios, when the writhe of the trefoil is the same as the handedness of the helical nanochannel—we call this system equichiral; in the second case, we investigate the case when the handedness of the channel is opposite to the writhe of the knot, calling this system antichiral. The knots were incorporated into the channels following the procedure described in the Methods section. The coordinates of the knots were generated by using parametric equations for a torus knot with *p* = 3 and *q* = 2, and then an arbitrary bond was interrupted, and the ends of the linear chain were pulled away from each other [27,91]. Once the knots were incorporated into the channel, the simulations were performed following the same procedure as described in the previous section on free knots. An external force *F*_ext_ = 0.05 ε_0_/σ = 0.07 pN colinear with the main axis of symmetry of the channel was applied to the beads.

**Figure 2:**
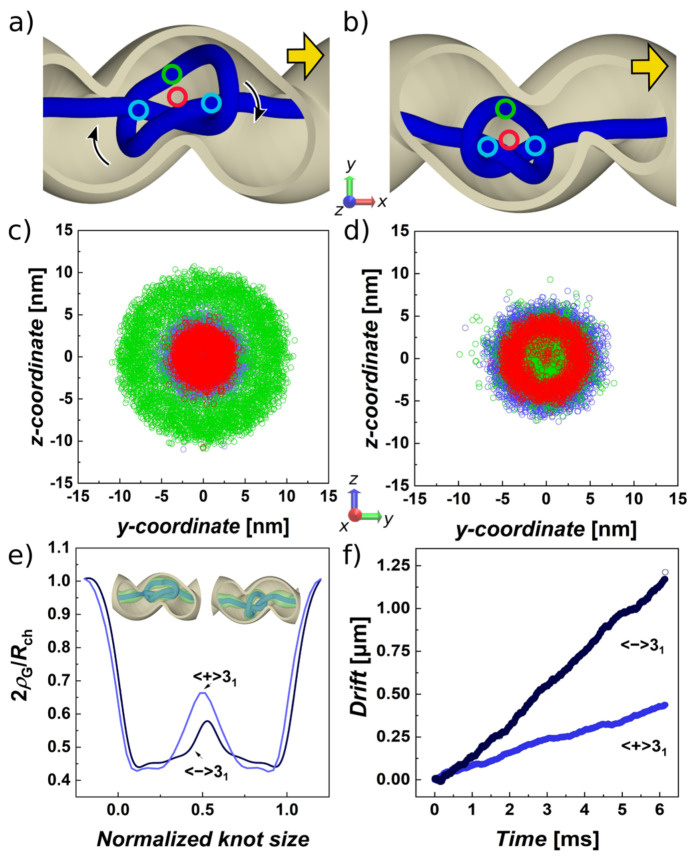
Trefoil knot in helical confinement. (**a**) A snapshot of trefoil with positive writhe <+>3_1_ in a channel with negatively oriented helicity representing an antichiral configuration. The blue circles indicate start and end bead of the knot detected by KymoKnot and Knoto-ID, the green circle indicates the middle bead of the knot, and the red circle is the center of mass of the knot. The yellow arrows indicate the direction of external pulling force for the particular snapshot. (**b**) Equichiral system of a trefoil with a negative writhe <−>3_1_ in a channel with negative helicity (**c**,**d**) distribution of the knot starts and ends middle points and center of masses of the knot. (**e**) Global radius of curvature (GRC) *ρ*_G_ compared to radius of the channel *R*_ch_ = 1.5 σ along the size of the knot; in the insets, the GRC is shown in green transparent color. (**f**) Total drift of the knot Δ*x* along the polymer as a function of time compared for the equichiral and antichiral systems.

The molecular dynamics trajectories of the equichiral and antichiral systems showed a striking difference of the movement of the confined trefoils. First of all, the trefoil in the antichiral configuration tends to be trapped by the grooves of the helical channel. As a result, the middle of the knotted portion, marked with green circle in Figure 2a, follows the helical groove and advances along the channel in the direction of the pulling force that is collinear with the main axis of the channel. In this situation, the arc of the knot orbits the center of the channel, where the crossings tend to localize. The crossings localize in the middle of the channel because of the twist induced by confinement (illustrated by arrows). This is illustrated also in Figure 2c, tracking the positions of different regions of the knotted portion marked as blue circles for the beads at the beginning and end of the knot with index *i*_start_ and *i*_end_, respectively; the middle of the knot is the bead *i*_middle_ = (*i*_start_ + *i*_end_)/2 depicted by a green circle; and the red circle shows the position of the center of mass of the knot. In the case of the equichiral system, when the sign of the writhe of the knot is equal to the handedness of the helix, the drifting of the knot along the helical channel occurs in a different manner, when the movement is more random and knot does not prefer moving in a particular conformation. The positions of the middle section of the knot and the beads denoting the knot’s start and end, and center of the mass overlap and their spread through the cross-section of the channel is much smaller and concentrated in a circle with the radius of about half of the total cross section of the channel, Figure 2d. The picture on the distribution of the selected characteristic points on the knot indicates that the knot in the equichiral configuration may be also much smaller as compared to antichiral confinement, Figure 2c,d. Hence, we also analyzed the geometrical measures of the knots in both scenarios. Table 2 indicates that gyration radii in helical confinement differ by 0.5 nm, while the difference is significant compared to the standard error deviation. The values of gyration radii and knot lengths are also lower than those obtained for the unconfined knots in free space, Table 1. The geometrical descriptors also indicate slightly more spherical shape of the knot in the equichiral confinement, while the knot also becomes more prolate as compared to unconfined knots as well as in the case of comparison with the values obtained for antichiral system.

In addition, in order to obtained a more detailed look on the differences between the shapes of the knots and distributions of the polymer forming the knot in the volume of the confining space, we adopted a calculation of the function called global radius of curvature [106]. This in simplicity calculates a maximum radius of a sphere, *ρ*_G_ (*x*), which can be rolled around a knotted portion of polymer chain of the length *x*∈ <0,*L*_k_ >. The calculation of the global radius of curvature yields a continuous profile in coordinates of the polymer curve, here conveniently expressed as beaded coordinates. The profile describes the local density of the molecule, which can represent the chain thickness; it can be related to writhe so it is a topological descriptor; and it is also related to internal energy [106]. Previously, we implemented *ρ*_G_ in order to analyze density of supercoiled molecules [107]. Here, we extend the definition of *ρ*_G_ for confinement so that the calculation of *ρ*_G_ is obtained not only from the beads forming the beaded chain, but it is also adjusted according to the nearest distance of confinement wall through analytical equations. The computed radii of curvature are shown in Figure 2e. The profiles are averaged over all structures in the five trajectories. The profiles start at value 2*ρ*_G_/*R*_ch_, meaning the unknotted chain tends to stay equally distant from the walls of the channel, in its center. Later, the *ρ*_G_ drops at the position of the crossings denoting the start (*i*_start_) and end (*i*_end_) of the knotted portion. The peak in the middle indicates the opening in the center of the cardioid structure of the knot. The peak is higher in the case of antichiral system, where the arc of the knot moves along the grooves of the helical channel.

Finally, in Figure 2f, we show the drift of the knot along the polymer as a function of time. The graph indicates that in the equichiral configuration, the trefoil with the negative writhe moves over twice as fast as the trefoil knot with a positive writhe. The corresponding values of *v*_drift_ and diffusivities *D* are shown in Table 2. The diffusivities were obtained as described in Section 3.1 of Results and Discussion. These parameters indicate stereospecific and selective behavior on the knotted superstructures with a given chirality.

### 3.3. Effect of Helical Radius, Pitch and Pulling Force on Separation of Knots

**Figure 3:**
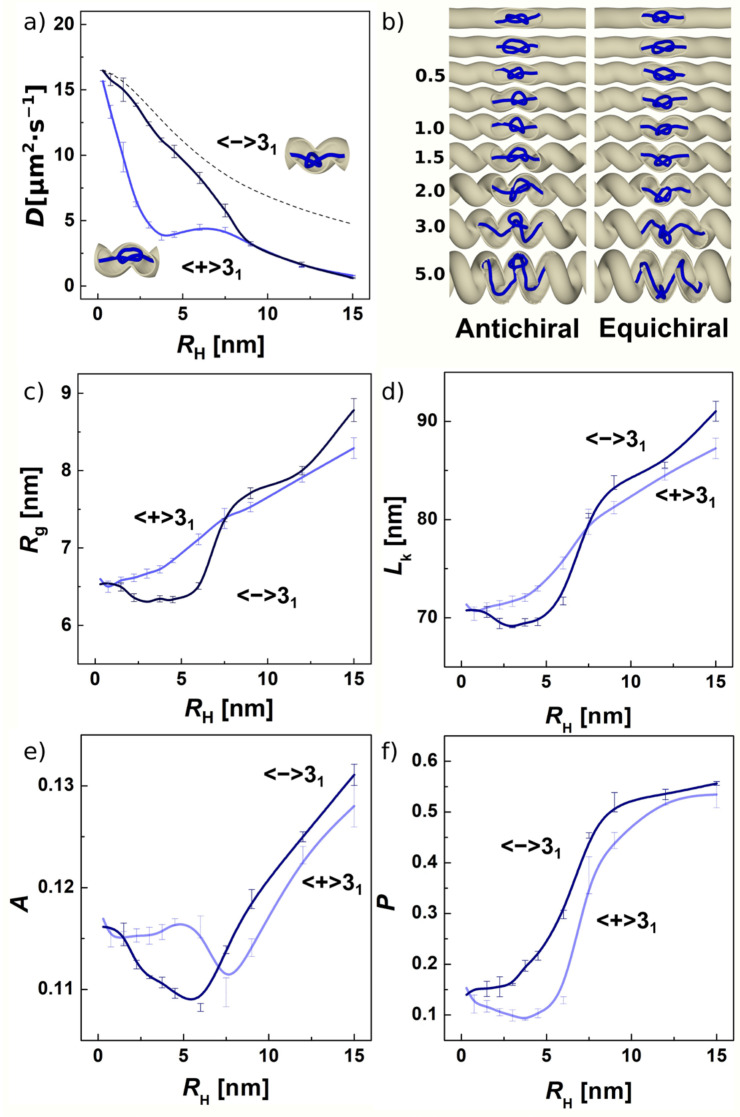
Effect of radius of the helix *R*_H_ on dynamic and geometrical properties. (**a**) Speed of self-reptation of righthanded <−>3_1_ and lefthanded knots <+>3_1_ placed in negatively wound helical channel (ω-) representing equichiral and antichiral system; dashed line corresponds to effective diffusivity predicted for a given tortuosity *D*_eff_ = *D*_0_/*τ* = *D*_0_*L*/*C*. (**b**) Snapshot of trefoils in helical channels for different radii of helix indicated by numbers. (**c**–**f**) Dependence of geometrical descriptors gyration radius, *R*_g_, knot length, *L*_k_, asphericity, *A*, and prolateness, *P*, with the radius of the helix.

In the next step, we investigate further the stereoselective effects observed in the previous section by varying parameters of the helix. As we described previously, aside from its chirality, the geometry of the helical channel is defined by three parameters, the channel radius *R*_ch_, the radius of the helix *R*_H_ and the pitch *k*; for illustration, see Figure S1. We determined the first parameter based on the size of the trefoil given by its gyration radii, *R*_g_, determined in the first section that investigates knots pulled in the free space. Secondly, the pitch of the helical channel, *k*, should not be smaller than a half of the *R*_ch_, so that the loops of the helix do not overlap or extend into each other that would make the cross-section of the channel larger. Hence, the parameter of our primary interest is the radius of the helix, *R*_H_. Investigating this parameter gives also a glimpse into the magnitude of the effect and how sensitive are the chiral superstructures to chiral modulation of the environment. In Figure 3a, the mobility of a trefoil knot and transport properties of the channel are shown in terms of diffusivities and the radius of the helix. The smaller values of the radius of the helix *R*_H_ mean the grooves in the helical channel are quite shallow with a small curvature, κ. On the other hand, when the radius of the helix exceeds the radius of the channel, the curvature κ increases, and the shape of the channels becomes more spiral like, where the term “spiral” would mean a spring-like object; see Figure 3b. 

The diffusivities in Figure 3a show distinct behavior between equichiral and antichiral systems. The diffusivities show a drop with different slope or intensity as a function of *R*_H_. The values of *D* are shown starting from a value of *R*_H_ = 0.1 σ = 0.3 nm. The values obtained at the smallest radius of the helix considered are 16.5 mμ^2^·s^−1^ and 15.5 mμ^2^·s^−1^. These values are slightly smaller than the diffusivity in the simulation of drifting knots in a free space *D* = 17 mμ^2^·s^−1^, as in Table 1. Since at the beginning, we chose the radius of the channel to be similar to the gyration radius of the freely moving trefoil, and the computed diffusivities start at the slightly lower values, meaning that the knots only start to feel the presence of the confinement. Interestingly, even with a tiny curvature κ of the helical channel, the knotted superstructures already start showing a systematic deviation from each other and sensitivity to the chiral environment. The diffusivities in the equichiral and antichiral configuration increasingly deviate from each other with an increasing radius of the helix. The maximum of the stereospecific sensitivity is reached for the radii of the helix *R*_H_ between 1 and 1.5 σ (3–4.5 nm), where the difference in mobility of over 200% is observed. Later, the diffusivities converge to the same value at *R*_H_ = 3σ = 9 nm, above which the radius of the helix becomes equal to and larger than the radius of the channel. Behind this point, the channel becomes spiral like, Figure 3b.

In practical situations, the decrease in the effective diffusivity with increasing radius of the helix of the confining channel can be justified by the increase tortuosity of the channel, τ [108]. Tortuosity is a common engineering parameter that in the simplest definition relates the ratio of the length of the curve travelled by a diffusing particle (*C*) to the distance between its ends *L*, *τ* = *C*/*L*. Then, the effective diffusivity is given as *D*_eff_ = *D*_0_/*τ*. Tortuosity of a helical channel can be expressed as *τ* = [(2π*k*)^2^ + (2π*R*_H_)^2^]^0.5^/2π*k*, where *k* stands for the pitch, and *R*_H_ is the radius of the helical channel. Tortuosity provides a good qualitative approximation of the change of effective diffusivity for equichiral system in the range of radii of the helical channel *R*_H_ < *R*_ch_, see Figure 3a (dashed line). For the antichiral system, i.e., trefoil with positive writhe in a negatively wound helix, and vice versa, we see differences that cannot be simply captured by a function of the curvature of space.

This infers that the diffusion of a knot in antichiral configuration is fundamentally different. The detailed picture on the situation of knot self-drifting in antichiral and equichiral configuration was provided in the previous section, where we showed that in the antichiral configuration the arc of the knot prefers to reside away from the center of the channel and proceeds along the channel by inducing an additional revolving motion in the grooves of the helical channel, Figure 2c. On the other hand, the knot in equichiral configuration shows that it is its center of mass that copies the curvature of the channel, Figure 2d, and hence, that is why there is a better agreement that the description of effective diffusivity by tortuosity, Figure 3a. It is intriguing to verify whether the slowdown of the self-diffusion in antichiral configuration cannot be a result of the revolving motion. For the effective diffusivity of a propelled chiral particle, a relation was derived *D*_eff_ = *D* + v_0_^2^ *D*_θ_/[2(D_θ_^2^ + *Ω*^2^)], [103,104]; here, *D*_θ_ is the coefficient of the rotational diffusion intrinsically bound with *D* since *D*_θ_ = 3*D*/(4*R*_H_^2^), *v*_0_ is the velocity induced by external force *F*_ext_*D*/ε_0_, and *Ω* is the speed of rotations, here induced by the helical channel with the pitch *k*, i.e., *Ω* = *v*_0_/(2πkσ). Since the effective diffusivity is a sum of rotational and translational contributions, the rotational motion can only be a result rather than a reason for the change of the diffusivity *D*. 

On studying drifting knots driven by electric currents, Di Stefano et al. proposed that the mobility of a particular knot type is related to the topological friction through a characteristic frictional length [73]. In confined space supposedly, this frictional length is increased by interactions of the knot with the walls of the channel. Since the spatial distribution of lefthanded and righthanded knots in chiral channel is different, this increases the topological friction imposed on the movement of knot. The helical geometry of the channels is relatively complex, posing challenges to envisioning the distribution of knot in the channel and how this interacts with the channel walls. We examine the distribution of the knot and interactions with the channel walls by different descriptors. First of all, Figure 3c shows the dependence of gyration radii on the radius of the helical channel *R*_g_ = *f*(*R*_H_). The computed data indicate that in the antichiral system the gyration radius of the knotted portion steadily grows. This indicates that the knot adjusts its radius and fills the loops of the helical channel, exerting more effective friction with the walls of the channel. In the case of the equichiral system, the knot maintains an approximately constant value of its radius to about value of *R*_H_~*R*_ch_, while the knot remains mainly in the middle opening of the channels.

The phenomenology reunites above *R*_H_ > *R*_ch_ where the effective diffusivity depends on tortuosity and also quantitatively the helical channel loses chiral sensitivity. A critical review on tortuosity approach pointed out its empirical character and the many definitions of tortuosity that exist [108]. Very recent works aimed on rigorous solutions of unbiased Brownian diffusion in helical channels provide analytical formulae for the case when the radius of the helix is larger than the radius of the channel, *R*_H_ >> *R*_ch_ [105,109,110]. Despite these works aiming to provide solutions for many problems in biology, our current simulations of the drifting knots in the helical confinement in terms of their diffusivities shown on Figure 3a and the various practical problems we reviewed in the Introduction indicate that the primary region of interest is around *R*_H_ ≤ *R*_ch_. It is also worthwhile to stress that tortuosity by itself is not expected to be sufficient to explain the deviations between the mobilities of different enantiomers. As remarked by Marenda et al., the topological sorting by channels with modulated geometry represents simple but yet underexplored mechanism [79]. In the past decades, a handful of works addressed a problem of particles diffusing through an entropy barrier [111,112,113,114,115]. The entropy barrier was imagined as a periodically bulging or diverging-converging chamber in a confining channel. In a practical scenario of proteins diffusing through nanopores, the barrier was modelled by a periodic potential [114]. Recently, the approach was extended for studying the diffusion of knotted molecules passing through a periodically modulated channel as passing through free energy profiles [79]. So far, the shape of the entropic barrier is represented by a periodic channel consisting of symmetrically rounded or conical chambers [111,112,113,114]. Using free-energy profiles for describing energy barrier to diffusing knots could be straightforward, as the global radius of curvature *ρ*_G_ is related to concentration, and hence the integral of the inverse of the *ρ*_G_ function is related to energy [106]. Nevertheless, solutions for arbitrary geometries would be difficult to solve [112].

**Figure 4:**
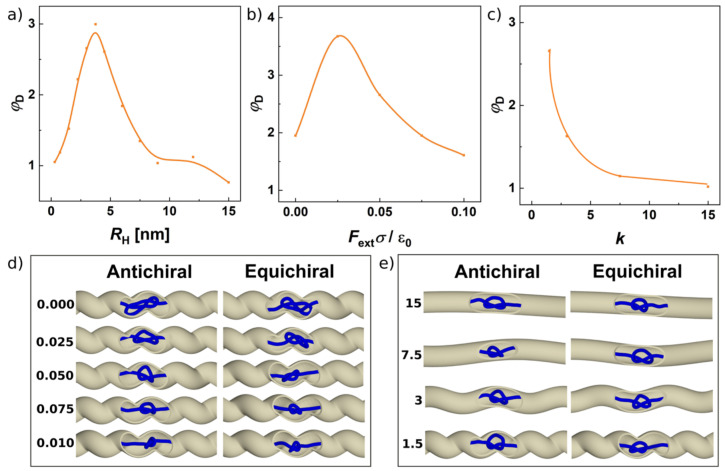
Stereoselectivity of the helical channel as a function of three parameters. (**a**) Effect of the radius of the helical channel on stereoselectivity of the channel in terms of *φ*_D_ = *D*_=_/*D*_±_. (**b**) Effect of the external pulling force *F*_ext_. (**c**) Effect of the distance between of the loops or steepness of the helical channel in terms of its pitch, *k*. (**d**) Snapshots from the simulations with varying external force, the values *F*_ext_σ/ε_0_ are indicated as numbers, for antichiral and equichiral configurations and channels with *R*_H_ = 1σ. (**e**) The channels with various pitch parameters, *k*, indicated as numbers to the left in helical channels with *R*_H_ = 1σ. The lines are provided as visual guides.

In addition, we investigate the influence of several operating parameters on stereoselective performance of the helical channel in terms of the ratio of the equichiral-to-antichiral diffusivities *φ*_D_ = *D*_=_/*D*_±_, as shown in Figure 4. First of all, on Figure 4a, we show summarized ratios *φ*_D_ as a function of the radius of the helix, *R*_H_. As we discussed earlier in Section 3.2, the ratio starts at the value of unity and increases with the increasing radius of the helix as two phenomenologically mechanism engage—one, where the knot is driven in the grooves of the channel revolving around the longest axis of the channel, and the second one, where the knot passes through the channel rather randomly as a fuzzy ball. Finally, the value of *φ*_D_ goes back to unity at *R*_H_ > *R*_ch_, inferring that there is an optimal value of the radius of the helical channel for the stereoselective performance of the channel between *R*_H_ = 1–1.5 σ, Figure 4a.

In Figure 4b, we show the ratios *φ*_D_ as a function of the external force applied. The range of applied forces was from the range of 0–0.14 pN. Again, the graphical dependence infers the existence of an optimal value of the external force, Figure 4c. When no external force is applied, the knots still show a difference in mobility induced by the chirality of environment. However, the diffusivities in absolute values are very small *D*_=_ = 0.30 μm^2^·s^−1^ versus *D*_±_ = 0.15 μm^2^·s^−1^. These values are similar to the values reported experimental for the pulling of knots on very long chains [21]. In our case, the reason of the small values of diffusivities in the presence of no external force is that the knotted portion is less localized, increasing the gyration radius of the knot, *R*_g_ = 9.2 nm, which generates more collisions with the walls of the channel, as seen in the snapshots in Figure 4d. The gyration radii decrease by increasing the strength of the external pulling force reaching *R*_g_ = 9.2, 7.5, 6.9, 6.0 and 5.7 nm for antichiral and *R*_g_ = 9.2, 6.9, 6.3, 6.0 and 5.7 nm for the equichiral system. When the pulling force is increased, the knot is much more localized, which decreases its radius of gyration what decreases collisions of the knot with the walls of the confining channel. As discussed in Section 3.1, the self-diffusivity of localized knots in free spaces should be more or less constant regardless the pulling force applied; however, the situation is more complex in the confined space, where the more localized knots exhibit fewer collisions with the walls of the confinement. Here, the knots stay localized not only by the effect of the pulling force but also because of the effects of confinement [71], but in the absence of the pulling force, the knots are more spread and exhibit more friction with the walls of the channel, which results in different and much smaller diffusivities. 

Finally, we investigated the effect of the pitch or the rise of the channel that defines the distance between the loops or the steepness of the helical channel, Figure 4c. Upon increasing the pitch of the helical channels, they lose their stereoselective capability in terms of *φ*_D_, since the density of the loops as the stereospecific features over the length of the channel decreases by increasing the parameter *k*. The dependence in Figure 4c shows the continuous drop of the value *φ*_D_ indicating that there is still a space for improvement of the stereospecific performance of the channel, and hence, the optimum size is yet to be reached. The setting of k where the highest *φ*_D_ is observed corresponds to length of the half period of the loop π*kσ* = 14 nm, which is by far sufficient to accommodating even the largest knots investigated. By decreasing the pitch *k*, however, the *φ*_D_ is expected to drop when the loops start to extend to each other.

From plenty of other parameters that may be of immediate interest to be explored with the observed stereosensitivity, such as polymer length, radius of the channels, *R*_ch_, electrostatics, explicit solvents, etc., which are not investigated here, we still considered it would be of most importance to test the proof of concept for a situation of non-nicked DNA. This is because of the phenomenon of revolving the knot in antichiral configuration observed in Section 3.2, Figure 2c. If the helical confinement induces the revolutions to the knot, this should be transferred to the chain and increase the torsional stress. This raises the question, whether the knot would be forced to leave its orbital trajectory and drift through the channel in the manner observed for equichiral system and the stereosensitivity would be lost. The simulations show that even if the modeled DNA chain possesses torsional stiffness, the stereosensitivity of the helical channels is maintained in terms of *φ*_D_ at the levels observed for nicked DNA, Figure S2. This could be attributed to the fact that the supercoiling diffuses away very fast but also that the chain length used in the simulations does not exceed 1 kbp (3 μm).

### 3.4. Different Chiral Knot Types Show Symmetry Breaking in Helical Channels, While Achiral Knots Do Not

The trefoil knot investigated so far is the most abundant of knots, with the highest probability of spontaneous formation on long linear chains [11,39,116], but it is also the most abundant knot found biologically on DNA and proteins. Despite this fact, more complex knots were also regularly found on DNA or prepared in experiments [117]. Hence, in the next step, we investigated the selectivity of channels with chiral modulation on more complex knots. The property of choice to represent different selectivity was again mobility of self-reptating knots in terms of drift speeds or diffusivities.

**Figure 5:**
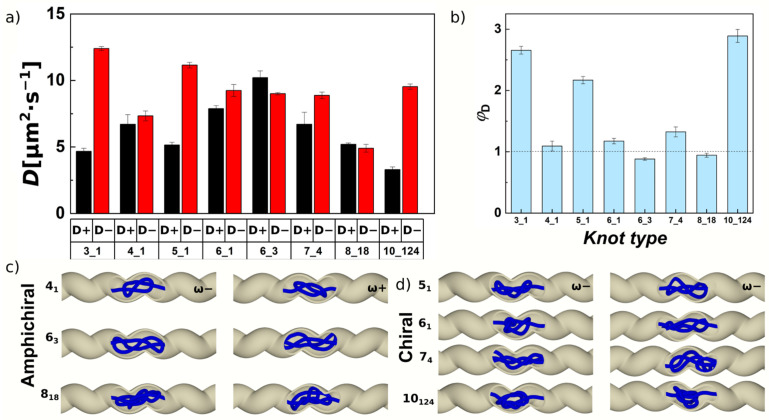
Stereoselectivity of helical channels (*R*_H_ = 1σ) for different knot types. (**a**) Diffusivities of grouped enantiomers of different knot types in helical confinement. For amphichiral knots (4_1_, 6_3_ and 8_18_), we flipped handedness of the channel rather than writhe of the knot. (**b**) Magnitude of the stereoselectivity of the helical channel expressed as *φ*_D_ = *D*_=_/*D*_±_. The dashed line indicates no selectivity. (**c**) Snapshots from simulations showing the situation of the knotted portions in helical channels for amphichiral system with righthanded (**left**) and lefthanded helical channel (**right**). (**d**) Snapshots from simulations showing the situation of the knotted portions in helical channels for chiral knots, with equichiral (**left**) and antichiral (**right**) placement of knots in righthanded helical channel. The handedness of the channels is indicated by ω+ and ω−.

In Figure 5a, we show the diffusivities grouped for given knot types for antichiral versus equichiral situation. The diffusivities by themselves show a complex behavior. This observation is in agreement with the previous studies of translocating knots, which showed that the topological friction of different knotoids do not exhibit a simple dependence on the knot type given by a crossing number [118]. Another study also showed that this dependence shows a complex behavior with varying external pulling force [119]. Figure 3b shows compilated data in terms of stereoselective performance of the channels given as *φ*_D_ = *D*_=_/*D*_±_. The data infer that the highest stereoselectivity is observed for torus knots (3_1_, 5_1_ and 10_124_), but a mediocre stereoselectivity is observed also for twist knots (6_1_ and 7_4_). Along with the chiral knots, we simulated also achiral knotoids with different complexity 4_1_, 6_3_ and 8_18_. During this investigation we varied the handedness of the channel rather than the sign of the writhe of the knots. The helical channels show no stereoselective effects on achiral knots yielding the value of *v*_D_ to be about unity. Figure 5c shows snapshots from the simulations of amphichiral and chiral knots.

### 3.5. Helical Channels Can Separate Chiral Knots Embedded on Circular Polymers

**Figure 6:**
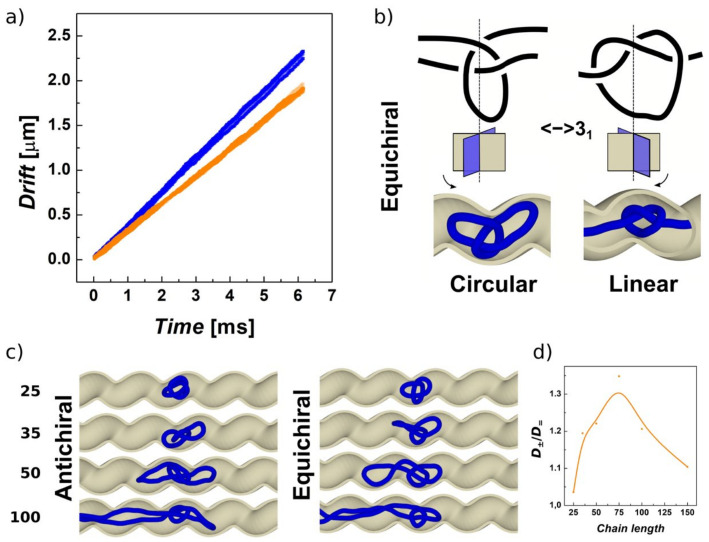
Stereoselectivity of helical channel (*R*_H_ = 1σ) on trefoil embedded on a circular chain. (**a**) Total drift over the time for antichiral and equichiral system shown for 5 overlapped trajectories for each setting. (**b**) Difference between orientation of the plane of the righthanded knot’s arc that is responsible for friction with the walls of the helical channel and stereoselective effect. (**c**) Snapshots from the molecular dynamics simulations showing circular chains with different handedness and also different lengths of the polymer indicated by number. (**d**) Relative difference between drift speeds in antichiral versus equichiral systems *D*_±_/*D*_=_.

In this part, we investigate the capability of helical confinement to sort knots embedded on circular chains based on their chirality. In this model, we used infinite channels with helical modulation periodically replicated through periodic boundary condition. Here, we used one directional pull with external force *F*_ext_ = 0.05ε_0_/σ whose direction did not alter. In Figure 6a, we show the drifting of the lefthanded and righthanded trefoil knots in antichiral and equichiral placement into righthanded channel as a function of time. The data shown in Figure 6a represent the overlapped values of the drift obtained from the five independent runs for each enantiomer. Based on the simulations, we observe a difference between the drift speeds of the enantiomers in chiral confinement. The observed difference between the drift speeds is about 20%, which is significant for confirming the stereoselective effect, even though the difference between mobilities is much lower than the stereoselective performance of the channels observed for knotoids reaching above 200%. The growing difference in drift exhibited by the enantiomers is shown in the Video S2 (lefthanded knot shown in green, righthanded in orange). 

However, the radius of the channels used in all simulations was the same and optimized for the gyration radius of the simplest of the knots localized under the pulling of the external force. In the case of knotted circular chains, these are topologically constrained even without the presence of the pulling force or confinement to induce a metastable knotted state. Hence, much larger channels could be used to exploit the chirality of the knotted superstructures for sorting of enantiomeric forms.

Secondly, we also observe that the stereoselective effect is inversed compared to knotoids. In the case of the circular chains, the helical confinement appears to affect knots in equichiral placement, i.e., a knot moves faster if the handedness of the channel is the opposite. The explanation of this phenomenon is that in the case of the circular chains, the knot favors a different configuration in the channel, where two chains run through the middle of the channel by extending an arc of the knot into the grooves of the channel. The plane of this arc twisted in an opposite direction compared to the situation encountered in the case of the knotoids, Figure 6b. 

The stereoselective effect shows a maximum when investigated for polymers with different chain lengths, Figure 6d. Here, the stereoselective effect computed as a ratio *D*_±_/*D*_=_. starts at value about 1, when the polymers are too small with a radius of gyration of about the radius of the channel. On the other hand, when the molecules become very long, the effect of the friction of one arc of a knot becomes relatively small to the overall friction of the polymer in the channel. The magnitude of the stereoselective effect is about 35% higher mobility of circular molecules of the length 75 σ (750 kbp). The magnitude of topological selectivity fits into a range of performance of periodically modulated channels, where it was observed for channels with periodic conical chambers that the variation of diffusivity between unknot and 41 knot ranges between 0% and 300%, depending on the length of the molecules. [79] The variation of relative diffusivity in modulated versus cylindrical channel *D*/D_0_ also showed a complex oscillatory behavior; however, much larger channels in terms of their radii were employed. [79] As we reviewed in the Introduction, the diffusion of knots in channels, translocations of knotted linear and circular chains, self-reptation of knotted chains, etc., were the subject of multiple works, and deeper investigation of the topological sorting in helical channels with varied radii would require a separate paper.

## 4. Conclusions

We took a journey into simulations of chiral knots and chirally modulated confining spaces in order to investigate whether knots on polymers could be recognized based on a simple topological sorting mechanism, i.e., by geometrically modulated channels represented here by helical tubes. 

At first, the simulations of knots drifting along the polymer chain were performed in the free space, and the trajectories were evaluated in terms of geometrical and dynamic parameters. These were comparable with the available data from computer experiments. The properties obtained in the free space were mirrored for the chiral enantiomeric pairs, thus also indicating that these cannot be used by themselves for distinguishing between the enantiomers. 

Later on, the knotoids were confined in helical channels with periodic geometrical modulation corresponding to helical shape. The helix was modelled as an Archimedean spiral maintaining the constant cross section of the channels as a function of the radius of helix, *R*_H_. Three parameters of the channels were scanned, the radius of the helix, the pitch and handedness of the helical channel. The helical channels showed a strong stereoselective effect on knotoids. This effect in terms of the drifting speed of diffusivity along the chain showed deviations by up to 200% between antichiral and equichiral configuration. The magnitude of the effect arises from the friction with the walls of the channel that is more effective in the antichiral configuration. This effect could be exploited by nanotechnological devices designed for the detection of the topological state of the knot. In our computer experiments, we kept one end of the chain anchored. We believe the setting is possible to achieve also by experiments, which often use anchoring of DNA molecules or employ stoppers to slow down the translocation of the DNA molecule through the channels. In addition, the setting with one end fixed corresponds to the biological situation when a knot is pushed along the DNA whether by molecular machinery or by supercoiling generated during transcription. The abundance of lefthanded knots observed experimentally could be related to slower drifting speeds of the knots in antichiral environment formed by negatively supercoiled DNA; this mechanism has to be tested in detail by a dedicated computational biology study.

Along with the design parameters of the helical channel, we also investigated the effect of the pulling force on the observed stereospecific effects. We saw that the dependence of stereospecificity shows a more complex behavior exhibiting a maximum in a range of tested pulling forces. This complex behavior arises from the fact that the pulling force affects the degree of localization of knots, while less localized knots exhibit large friction with the walls of the channel. Because of the increased friction the diffusivity of enantiomers drops, however, the stereoselective effect is observed to be maintained even in the absence of the pulling force.

Besides the trefoil knot, which is the most abundant of knots that can occur probabilistically on long polymer chains or in biological systems on DNA, we also investigated the magnitude of stereoselectivity of the helical channels for more complex knots. Our computer simulations indicate that the stereoselective properties of helical channels can be exploited also for probing the chirality of very complex knots. The simulations show also that achiral knots are not sensitive to the channels whether the handedness of knots or channels is mirrored.

Finally, we investigated whether the observations of the chiral selectivity of helical channels apply also on circular knotted chains. So far, the simulations have shown that the stereoselectivity is weaker, but the channels were optimized for knotoids in terms of the radius of the channels. The observed effect is strong enough to be exploited experimentally. 

## Figures and Tables

**Table 1:** Geometrical and dynamic measures of the knotoids. The first column indicates the knotoid name in terms of the convention implemented in the Knoto-ID software [46]. The second column indicates the simplified name of the knot, with the sign corresponding to the sign of writhe; Δ*Wr* is the mean value of writhe [100,101]; *R*_g_ is gyration radius; *L*_k_ is the contour length of the knot; *A* is the asphericity; *P* is the prolateness [99]; *v*_drift_ and *D* are dynamic properties, drifting speed and diffusivity. Bottom row also shows representative conformations with their ellipsoids of inertia with the main axes of inertia used for calculating *A* and *P*.

Knotoid	Knot	Δ*Wr*	*R*_g_	*L*_k_	*A*	*P*	*v*_drift_	*D*
			[nm]	[nm]			[μm·s^−1^]	[μm^2^·s^−1^]
3_1m	<+> 3_1_	3.27	7.7 ± 0.1	79.5 ± 0.5	0.11	−0.32	312 ± 14	16.94
3_1	<−> 3_1_	−3.27	7.8 ± 0.1	80.1 ± 0.6	0.11	−0.33	316 ± 5	17.58
4_1|11n_19	4_1_	0	9.0 ± 0.0	102.6 ± 0.2	0.16	0.41	239 ± 7	13.05
5_1m|10_132m	<+> 5_1_	6.1	8.6 ± 0.0	115.1 ± 0.0	0.09	−0.36	307 ± 5	15.63
5_1|10_132	<−> 5_1_	−6.10	8.9 ± 0.1	117.6 ± 0.0	0.09	−0.39	302 ± 5	16.79
5_2|11n_57m	<+> 5_2_	4.56	10.6 ± 0.1	128.9 ± 0.9	0.17	0.69	304 ± 15	17.08
|12n_475m								
5_2m|11n_57	<−> 5_2_	−4.57	10.9 ± 0.5	132.3 ± 4.3	0.16	0.67	302 ± 7	16.71
|12n_475								
6_1	<+> 6_1_	1.17	12.8 ± 0.1	158.0 ± 0.6	0.21	0.84	265 ± 6	14.68
6_1m	<−> 6_1_	−1.18	12.5 ± 0.2	156.8 ± 2.6	0.2	0.77	276 ± 16	15.22
7_1m|12n_749	<+> 7_1_	8.77	11.5 ± 0.1	172.3 ± 4.5	0.1	0.14	334 ± 7	19
7_1|12n_749m	<−> 7_1_	−8.82	11.3 ± 0.1	164.5 ± 4.5	0.12	0.37	313 ± 7	17.39
10_124	<+>10_124_	10.7	10.0 ± 0.3	167.9 ± 3.5	0.07	−0.42	359 ± 6	18.26
10_124m	<−>10_124_	−10.71	9.8±0.3	161.8 ± 3.5	0.07	−0.41	347 ± 6	19.61
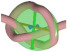 3_1m	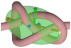 4_1|11n_19	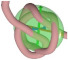 5_1| 10_132		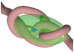 5_2|11n_57m|12n_475m	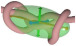 6_1		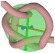 7_1m| 12n_749	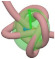 10_124

**Table 2:** Geometrical and dynamic measures of the knotoids confined in a helical tube with radius RH = 1.5σ, *R*_ch_ = 3 σ and pitch *k* = 1.5 σ.

System	Knot	Δ*Wr*	*R*_g_ [nm]	*L*_k_ [nm]	*A*	*P*	*v*_drift_ [μm·s^−1^]	*D* [μm^2^·s^−1^]
Antichiral	<+>3_1_ (m)	3.24	6.8 ± 0.0	73.0 ± 0.3	0.12	0.10	87 ± 4	3.94 ± 0.22
Equichiral	<–>3_1_	−3.43	6.3 ± 0.0	69.6 ± 0.4	0.11	0.22	188 ± 5	10.29 ± 0.48

## Data Availability

All data presented in this study are available on request from the corresponding author. The data are not publicly available due to the extensive size of the MD trajectories.

## Supplementary Materials

The following are available online at https://www.mdpi.com/article/10.3390/polym13213726/s1, Figure S1: The helical channel, Figure S2: Model of a knotted linear DNA chain with torsional stiffness; Figure S3: Movie strips of trefoils in helical confinement, Video S1: Molding the channel, Video S2: The knot race.
