# Supplementary material for: Channels with Helical Modulation Display Stereospecific Sensitivity for Chiral Superstructures
Source: Polymers (Basel). 2021 Oct 28;13(21):3726. doi: 10.3390/polym13213726 (PMC8588256; doi:10.3390/polym13213726)
Supplement: Supplementary file 1 [file polymers-13-03726-s001.zip › Supplementary.pdf]

`set pi 3.141592653`	π
`set Nbc 0`	Explicit particles
`set Umax 18`	Beads forming circles
`set Vmax 180`	Longitudinal beads
`set k 1.5`	Pitch, k
`set rh 1.5`	Radius of helix, R_H
`set rch 3.0`	Radius of channel, R_{ch}
`set Az [expr $k / pow($k*$k+$rh*$rh,0.5)]`	
`set Ay [expr $k / pow($k*$k+$rh*$rh,0.5)]`	
`set Ax [expr $rh / pow($k*$k+$rh*$rh,0.5)]`	
`for {set i 1} { $i <= $Umax } { incr i } {`	
`  for {set j 1} { $j <= $Vmax } { incr j } {`	
`    set u [expr 2.*$pi/18.*$i]`	Cross-sectional angle
`    set v [expr 2.*$pi/(1.0*$Vmax/(1.0*$nloops))*$j]`	Period of the helix
`    set z [expr ($Az*$rch*sin($u)*cos($v)-$rch*cos($u)*sin($v)-$rh*sin($v))]`	Z coordinate
`    set y [expr ($Ay*$rch*sin($u)*sin($v)+$rch*cos($u)*cos($v)+$rh*cos($v))]`	Y coordinate
`    set x [expr ($Ax*$rch*sin($u)+$k*$v*(10.0/$nloops))]`	X coordinate
`    if {$j%1==0} then {`	
`     set Nbc [expr $Nbc+1]`	
`     part [expr $Nbc+$NPART*6] pos $x $y $z type 2 fix 1 1 1`	Generate beads in ESPResSo MD
`    }`	
`  }`	
`}`	

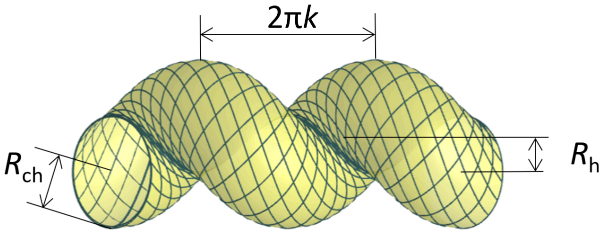

$$x = \frac{R_H^2}{(k^2 + R_H^2)^{0.5}} R_{ch} \sin(u) + kv$$

$$y = \frac{k}{(k^2 + R_H^2)^{0.5}} R_{ch} \sin(u)\sin(v) + R_{ch}\cos(u)\cos(v) + R_H \cos(v)$$

$$z = \frac{k}{(k^2 + R_H^2)^{0.5}} R_{ch} \sin(u)\cos(v) - R_{ch}\cos(u)\sin(v) - R_H \sin(v)$$

Figure S1. The helical channel. A tcl/tk script to generate surface particles of confining helical channel with chosen parameters of radius of the channel, R_{ch}, radius of the helix, R_H, and the pitch, k. The script generates in default setting $N_{loops} = 10$ a helix with ten loops. If a different setting of N_{loops} is used, the resulting pitch will be given as $V_{max}/(U_{max}*N_{loops})$. At the bottom, a schematic drawing shows resulting surface with the meaning of the parameters. Additionally, parametric equations implemented in the script are shown in mathematical transcription.

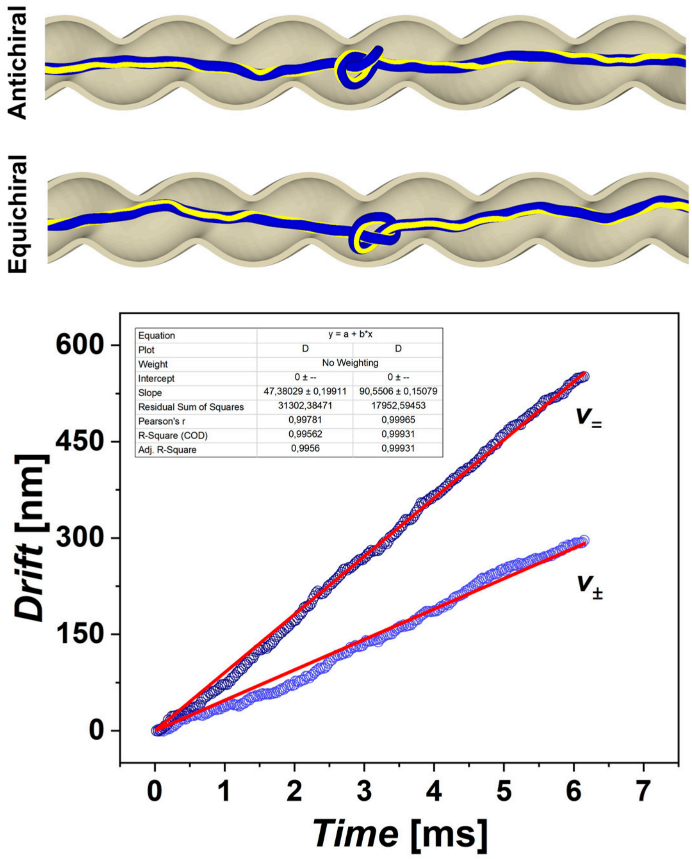

Figure S2. Model of a knotted linear DNA chain with torsional stiffness placed in a helical channel with R_{ch}=3σ, R_H=1σ and k = 1.5σ. Here, the chain with the torsional stiffness cannot freely rotate around the axis and represents the DNA in non-nicked state. The torsional stiffness was incorporated by adding additional particles to the chain, that are attached periaxially to the main chain. Consecutive pairs of the periaxial beads are bound together by a dihedral potential. The full description of the model and parametrizations can be found in the Ref.'s [1-4]. The snapshots from the simulations are shown in the upper part of the Figure S2. The main chain is shown in blue, with yellow stripes indicating distribution of twist along the chain that also correspond to positions of the periaxial beads. Since drifting of the knotted portion of polymer is associated with rotational motion (Section 3.2), this revolving motion of the knot along the chain could be responsible for generating an axial stress. As the snapshots indicate, we do not see a substantial accumulation of the torsional stress of supercoiling. The axial stress was shown earlier to diffuse very quickly along the DNA, being released at sinks represented by the ends of the chain, nicks, or topoisomerases [5,6]. However, the future works might want also to take in account the length of the DNA chains that in the current study does not exceed 1 kbp (3 μm). In the bottom part, the total drift as a function of time obtained from averaging 5 trajectories for equichiral (=) and antichiral (±) systems is shown. The drift speed corresponds to the slope of the total drift versus time. The values of drift speeds obtained by fitting indicate, that the knots move about twice faster in equichiral configuration than in the antichiral system. This is comparable with the value obtained for the same geometry of the channel and the nicked DNA, where we reported drift speeds to be around 2.2 times faster for equichiral system (Figure 4a). This suggests, that the stereosensitive effect of the helical channel would apply also for non-nicked DNA.

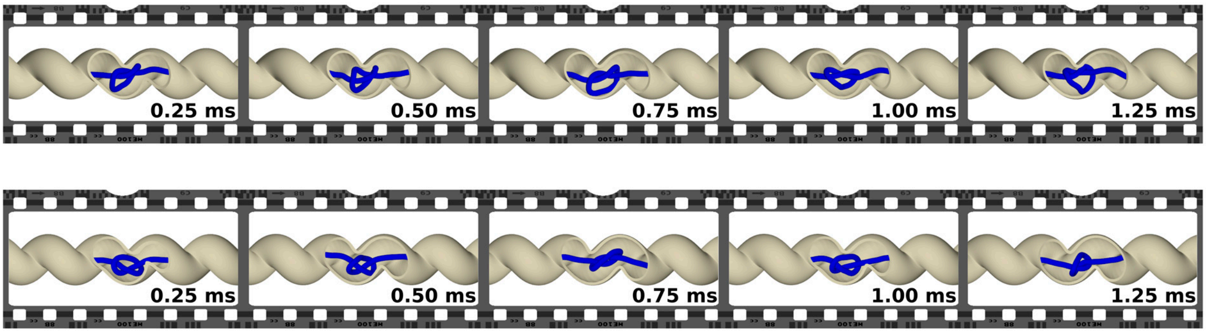

Figure S3. Consecutive snapshots from a molecular dynamics trajectory separated by 0.25 ms obtained by simulations of a trefoil in helical confinement with antichiral setting of handedness of the knot versus the handedness of the helical winding, shown in the top row; in the bottom row, a short trajectory is shown for equichiral configuration of the system, i.e. righthanded trefoil confined within a righthanded helical channel.

References

1. Racko, D.; Benedetti, F.; Dorier, J.; Burnier, Y.; Stasiak, A. Molecular Dynamics Simulation of Supercoiled, Knotted, and Catenated DNA Molecules, Including Modeling of Action of DNA Gyrase. *Methods in molecular biology* **2017**, *1624*, 339–372.
2. Racko, D.; Benedetti, F.; Dorier, J.; Burnier, Y.; Stasiak, A. Generation of Supercoils in Nicked and Gapped DNA Drives DNA Unknotting and Postreplicative Decatenation. Nucleic Acids Research **2015**, 43, 7229–7236.
3. Benedetti, F.; Japaridze, A.; Dorier, J.; Racko, D.; Kwapich, R.; Burnier, Y.; Dietler, G.; Stasiak, A. Effects of Physiological Self-Crowding of DNA on Shape and Biological Properties of DNA Molecules with Various Levels of Supercoiling. *Nucleic Acids Research* **2015**, *43*, 2390–2399, doi:10.1093/nar/gkv055.
4. Benedetti, F.; Racko, D.; Dorier, J.; Stasiak, A. Introducing Supercoiling into Models of Chromosome Structure. In Modeling the 3D Conformation of Genomes; CRC Press: Boca Raton, FL, USA, **2019**; pp. 115–138.
5. Koster, D.A.; Crut, A.; Shuman, S.; Bjornsti, M.-A.; Dekker, N.H. Cellular Strategies for Regulating DNA Supercoiling: A Single-Molecule Perspective. Cell **2010**, 142, 519–530.
6. Rusková, R.; and Račko, D.; Entropic Competition between Supercoiled and Torsionally Relaxed Chromatin Fibers Drives Loop Extrusion through Pseudo-Topologically Bound Cohesin; Biology **2021**, 10(2), 130; https://doi.org/10.3390/biology10020130
